# Antisense oligonucleotides for Alzheimer's disease therapy: from the mRNA to miRNA paradigm

**DOI:** 10.1016/j.ebiom.2021.103691

**Published:** 2021-11-10

**Authors:** Wioleta Grabowska-Pyrzewicz, Andrew Want, Jerzy Leszek, Urszula Wojda

**Affiliations:** a Laboratory of Preclinical Testing of Higher Standard, Nencki Institute of Experimental Biology of Polish Academy of Sciences, Pasteur 3, 02-093, Warsaw, Poland; b Department of Psychiatry, Wroclaw Medical University, Wybrzeże Pasteura 10, 50-367 Wroclaw, Poland

**Keywords:** Neurodegeneration, Alzheimer's disease, therapy, gene therapy, RNA therapy, antisense oligonucleotide, miRNA, RNA delivery, preclinical testing, clinical trials, AD, Alzheimer's disease, Aβ, Amyloid beta, APP, Amyloid precursor protein, ASO, Antisense oligonucleotide, BBB, Blood-brain barrier, CSF, Cerebrospinal fluid, NFT, Neurofibrillary tangle

## Abstract

Alzheimer's disease (AD) represents a particular therapeutic challenge because its aetiology is very complex, with dynamic progression from preclinical to clinical stages. Several potential therapeutic targets and strategies were tested for AD, in over 2000 clinical trials, but no disease-modifying therapy exists. This failure indicates that AD, as a multifactorial disease, may require multi-targeted approaches and the delivery of therapeutic molecules to the right place and at the right disease stage. Opportunities to meet the challenges of AD therapy appear to come from recent progress in knowledge and methodological advances in the design, synthesis, and targeting of brain mRNA and microRNA with synthetic antisense oligonucleotides (ASOs). Several types of ASOs allow the utilisation of different mechanisms of posttranscriptional regulation and offer enhanced effects over alternative therapeutics. This article reviews ASO-based approaches and targets in preclinical and clinical trials for AD, and presents the future perspective on ASO therapies for AD.

## Graphical abstract

**Figure:**
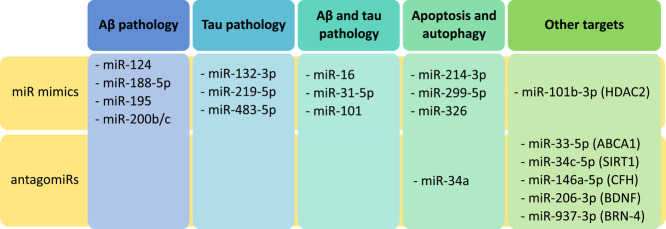

## 1 Introduction

Alzheimer's disease (AD) is an irreversibly progressing, ageing-related neurodegenerative disorder affecting over 55 million people worldwide and the cause of 60-70% of dementia cases. By 2050 the number of AD patients is predicted to triple, making AD one of the major social threats of the 21st century given the complete absence of disease-modifying therapies [1]. Currently AD is viewed as a progressing biological and clinical continuum. It starts within the temporal area of the brain in the hippocampus and is marked by extracellular deposits of Aβ peptides (senile plaques) and intraneuronal neurofibrillary tangles (NFTs) formed by hyperphosphorylated tau protein. Gradually AD progresses to the cerebral cortex and other brain areas and clinically manifests as progressing memory loss and cognitive impairment.

Therapeutic approaches tested for AD can be classified as cell-based therapies, including stem cells or bone marrow transplants, and molecular therapies targeted to proteins or nucleic acids. Proteins are usually modified using antibodies or protein inhibitors, or replaced by synthetic proteins in enzyme replacement therapies. Gene therapy methods are aimed at correcting mutations in DNA using viral or plasmid vectors. In turn, therapies targeting RNA are developed mainly based on synthetic antisense oligonucleotides (ASOs), small synthetic molecules designed to regulate protein translation.

The first step in the design of all targeted molecular therapies is identification of proper target(s) and their tissue and/or cellular location as a result of the investigation of pathways and molecules involved in a pathology. A review of more than 2000 interventional clinical trials of potential drugs for AD (closed or ongoing) shows that the dominant ones are based on the amyloid cascade hypothesis [2,3]. More than 260 clinical trials have targeted the amyloid beta (Aβ) peptide known to accumulate in the AD brain in the form of extracellular senile plaques. These result from faulty processing of transmembranous amyloid precursor protein (APP), first by β-secretase BACE1 and later by a complex of γ-secretase containing presenilin 1 or presenilin 2 (PS1, PS2) as enzymatic core. Immunotherapy with anti-Aβ antibodies was considered the likeliest strategy, recently exemplified by FDA accelerated approval for aducanumab (Biogen) which still requires verification of the expected clinical benefits. Amyloidogenic proteolysis of APP has also been targeted using BACE1 or PS1 inhibitors as potential therapeutics. Another popular target is microtubule associated tau protein (MAPT, tau) which, upon hyperphosphorylation by GSK3β, ERK1, CDK5, and some other kinases, is known to be deposited inside neurons in the form of neurofibrillary tangles (NFTs) and to impair axonal transport. Clinical trials targeting only tau protein as well as both tau and Aβ have been started, whereas the number of clinical trials aiming at targets other than Aβ or tau represent only about 1% [3]

Amyloid hypothesis-centred approaches in the drug development process originate from studies on the hereditary, early-onset, familial form of AD (FAD), that accounts for about 1% of AD incidences. FAD is predominantly caused by mutations in genes encoding APP, PS1, or PS2. It is now realised that the aetiology of sporadic, late-onset AD (SAD) is much more complex, with long-lasting, ageing-dependent dynamic development and progression from latent preclinical to clinical stages, modified by environmental risk factors [4,5]. It seems that ageing-related metabolic and systemic low-grade inflammation can trigger AD by blood-brain barrier (BBB) impairment and induction of neuroinflammation. At the cellular level, AD pathogenesis is currently seen as a result of several ageing-related molecular mechanisms which comprise, but are not limited to, loss of proteostasis, lipid dyshomeostasis, genomic instability, calcium dyshomeostasis, and mitochondrial and oxidative stress [6,7]. AD, therefore, represents a particular therapeutic challenge. All AD clinical trials have failed to produce the expected results, questioning the applied therapeutic paradigms.

What are the lessons from the clinical trials conducted so far? First, increased specificity of targeting is required to lower side effects of treatments. Second, choice of therapeutic targets should be adjusted to the disease stage and subtype. Third, AD as a multifactorial disease seems to require multi-targeted therapies, affecting several aspects of pathology. Fourth, once the therapeutic target(s) and its cellular/tissue location is established, the protocol for delivery of therapeutic agents has to be selected to ensure their efficient delivery to the right place and at the right time. The effective delivery of therapeutic agents, protected from degradation, to desired cells, with circumvention of the BBB and cell membrane to access subcellular targets is one of the major challenges to overcome for successful therapy.

Targeting mRNA with short, synthetic, antisense oligonucleotides (ASOs) might help to respond to the above requirements. ASOs’ complementary binding with RNA based on Watson-Crick base pairing is of high specificity. Moreover, the technological advancements in the methods for ASO delivery to the brain have paved the way for their development as therapeutics in neurological diseases [8], [9], [10]. In addition, discovery in 1990 of RNA interference, and of non-coding microRNAs (miRNA), opened exploration of miRNAs as novel therapeutic targets for ASOs. In the next chapters we compare strategies of mRNA and miRNA regulation using ASOs and review ASOs which proved successful in cellular and animal AD models. The ASO mimics illustrate that RNA can also play a vital effector function in molecular targeted therapies, whilst antagomiRs can target RNA.

## 2 Classical paradigm of molecular targeted therapies

**Fig. 1:**
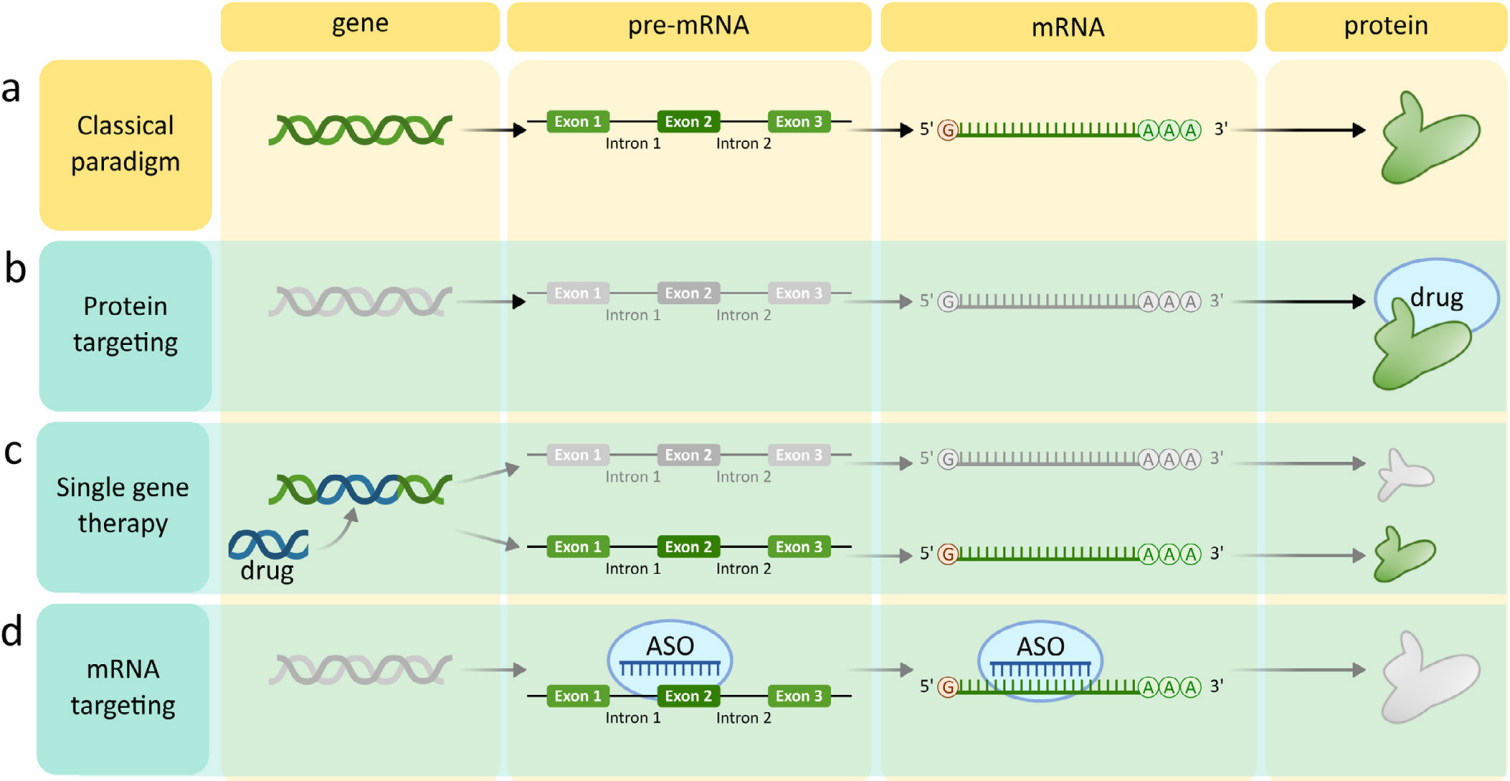
**Classical paradigm on flow of biological information and related targeted therapeutic strategies.** (a) Scheme of the fundamental paradigm of biological information flow. (b & c) Annotation of biological information flow with site of traditional drug action. (d) Highlighted site of action for ASO drugs.

Except for some biological systems, such as RNA-viruses, a fundamental paradigm in biology assumes the flow of genetically encoded information from DNA (gene) to messenger RNA (mRNA, transcript) to protein (effector), implementing specific biological functions. This paradigm defined the three types of therapeutic targets: genes, RNA transcripts, and proteins (Fig. 1a). Currently**,** the vast majority of existing drugs are small molecule therapeutics or antibodies which affect activity of pathologically altered proteins, including the products of mutated and dysfunctional genes (Fig. 1b). Gene therapy (Fig. 1c), marked by the successful completion of the first treatment for adenosine deaminase deficiency in 1990, proved to be more difficult, with only very few gene therapy-based drugs currently marketed. The main challenges faced by gene targeting are: efficient and inflammation-risk-free gene delivery to desired tissues and cells in human organisms, protection from degradation, prevention of a therapeutic gene insertion at undesired sites in the genome resulting in tumorigenicity, and proper control of the gene expression level. Although these problems have not been fully solved, the gene therapy approach stimulated significant development of nucleic acid delivery methods to human tissues, mainly using viral or non-viral vectors. These methodological advancements inspired approaches directed to mRNA regulation using ASOs (Fig. 1d).

## 3 mRNA regulation with ASOs

**Fig. 2:**
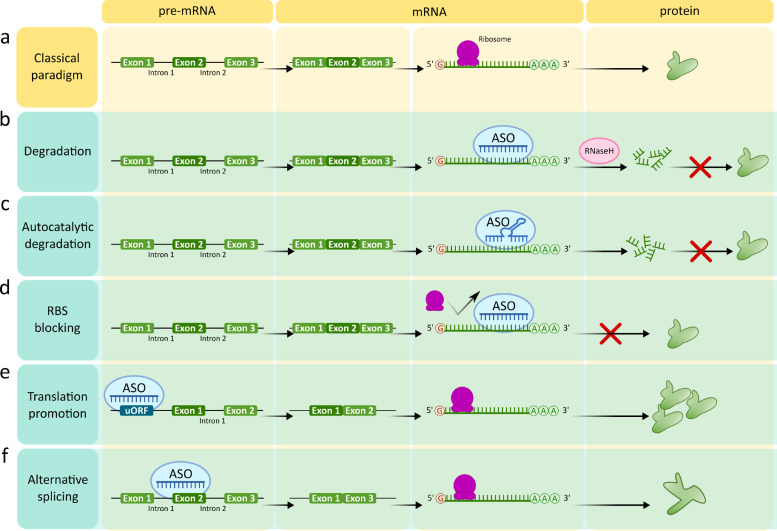
**Strategies for mRNA and pre-mRNA regulation using ASO.** (a) Scheme of the fundamental paradigm of biological information flow. (b) RNase H-triggered mRNA degradation. (c) Ribozyme-mediated autocatalytic degradation. (d) Steric blocking of ribosome binding site. (e) Translational promotion by upstream ORF binding. (f) Promotion of alternative splicing.

ASOs directed to mRNA as synthetic single-stranded DNA molecules received an additional dimension for therapy with the discovery of RNAse H-mediated degradation of RNA-DNA hybrids [11,12].

(Fig. 2b). The maximal length of ASOs was defined as under 30 nucleotides because longer ASOs proved to be unstable *in vivo*. Then followed ribozymes and DNA-zymes, RNA or DNA ASOs with intrinsic nuclease activity intended for direct cleavage of a target mRNA [13,14] (Fig. 2c). A proof of concept was also obtained for RNA ASOs which were designed either to decrease mRNA translation by means of a steric block of ribosomal binding sites or 5’ cap binding (Fig. 2d), or to increase translation by binding to specific regulatory sequences such as upstream open reading frame (uORF) (Fig. 2e). ASOs have also been designed for modulation of pre-mRNA splicing (Fig. 2f). ASO-mediated inclusion or exclusion of particular exons enables reinstatement of proper splicing and restoration of wild-type protein expression, exclusion of mutated fragments, a shifted ratio of splicing variants, or introducing out-of-frame deletion resulting in mRNA nonsense decay [15]. For realisation of all ASOs-based therapeutic strategies, it is critical that they are delivered to the correct location whilst minimising immunogenicity, degradation, or inactivation. Such methods are reviewed in the section *3.1. ASO modifications and delivery strategies for therapeutic applications* and elsewhere [[8], [9], [10],^16^].

### 3.1 ASO modifications and delivery strategies for therapeutic applications.

Upon administration, ASOs need to travel through blood or cerebrospinal fluid (CSF), to reach their cellular destination. For therapy of brain diseases, ASOs must cross the BBB cell membranes and then the membranes of the brain cells. Next, ASOs must resist or escape intracellular degradation mechanisms, mainly by endogenous nucleases. Several strategies are still being optimised to provide the best route of ASO administration, to improve ASO stability, lower immunogenicity, and to enable efficient delivery and internalisation of ASOs in specific cells. Three approaches predominate for the effective delivery of ASOs: direct chemical modification of the ASO molecule, conjugation with specific targeting molecules, and encapsulation in non-viral vesicles.

*Chemical modifications*

Three generations of chemically modified ASOs exist.

• 1st: a non-bridging oxygen atom of the inter-nucleotide phosphate group is replaced with a sulfur atom which creates a phosphorothioate (PS) backbone, increasing nuclease resistance.
• 2nd: in addition to PS backbone, a ribose sugar is modified which further improves nuclease resistance and binding affinity. Such ASOs are designed as GAPmers in which central PS nucleotides are flanked on both sides with sugar-modified nucleotides or as MIXmers in which PS nucleotides are interspaced with sugar-modified ones. GAPmers combine efficient RNase H1 recruitment with improved endosome resistance and binding affinity.
• 3rd: further modifications such as a) locked nucleic acids (e.g. restrictive bridging of 2’ and 4’ carbons of ribose) which increase RNA affinity but block recruitment of RNase H unless included in GAPmers or MIXmers; b) nucleobase modifications (such as methylation on 5’ cytosines, 2-O-methoxyethyl (MOE) or constrained ethyl (cEt) groups [17]) which improve target binding; c) alternative backbones (phosphorodiamidate morpholino backbone, P-ethoxy backbone - ethyl group added to a non-bridging oxygen atom in phosphate backbone, pseudo-peptide polymer backbone) which increase stability, degradation resistance, target affinity and reduce toxicity.

*Conjugation*

While chemical modifications render ASOs more stable, they also decrease their ability to pass cell membranes. To improve ASO cellular uptake and targeting specific cell-types, ASOs are conjugated to molecules with affinity to particular cell membrane proteins such as:

• Receptor ligands or antibodies which enable cell entrance of the ASO conjugate via receptor-induced endocytosis.
• Aptamers/chemical antibodies (oligonucleotides or peptides) forming 3-dimensional structures interacting with surface proteins similarly as antibody-antigen complexes and facilitating internalization. Aptamers show low immunogenicity, are easy to design and inexpensive.

*Delivery in non-viral vectors*

Increase in efficiency of ASO delivery, protection from degradation, shield the negative charge for more efficient cellular uptake and lower immunogenicity can be achieved with encapsulation of ASOs in non-viral vectors of several types:

• Cationic polymers of synthetic or natural origin, usually biocompatible and biodegradable, such as PLA (polylactic acid), PLGA (poly(lactic co-glycolic acid)), or PEI (Polyethyleneimine).
• Lipidic vesicles, mainly liposomes - phospholipid bilayer vesicles formed in aqueous solutions that easily penetrate the cell membrane. They can carry drugs that are hydrophilic (in the core) or hydrophobic (in the lipid bilayer). They differ in size, lipid composition, and modifications. An alternative are exosomes or apoptotic bodies naturally secreted by cells.
• Inorganic nanoparticles - conjugates of ASOs with magnetic or gold nanocarriers (gold nanocages), or with graphene-based or silica-based nanoparticles known as quantum dots (QDs). QD are semiconductor crystals that offer the possibility of tracing delivery with bioimaging.

Non-viral vectors can be additionally functionalised for selective delivery by adding surface ligands for specific receptors on the target cells. Coating of ASO carriers with polyethylene glycol (PEG) is known to lower their immunogenicity.

*Administration strategy*

The route of administration can influence distribution, targeting, and accumulation of therapeutic ASOs at the site of action. In the non-conjugated form ASOs are water-soluble, easily formulated in phosphate buffers and administered subcutaneously, intravenously, intraperitoneally, or directly to the site of action. In clinical studies concerning central nervous system diseases ASOs are often administered to the spinal cord (intrathecally) and then distributed via CSF. It was shown that ASO half-life is extended in the CSF (in comparison to the periphery) [18].

*ASOs approved for clinical use*

All eight ASOs approved as drugs rely only on chemical modifications for stability improvement. Half of the eight clinically used ASOs are injected intravenously, the other four are administered either subcutaneously or directly into the site of action: eye (intravitreally) or intrathecally. It was shown that following intrathecal administration, ASOs are efficiently distributed within CSF and reach the brain, spinal cord and cortical tissue [19,20]. They are then cleared into plasma producing almost untraceable levels [21]. This delivery approach was already successfully implemented in clinical trials for treatment of neurodegenerative diseases [22,23] and shows that ASOs can be effective therapies when combined with appropriate delivery methods.

## 4 ASOs regulating mRNA in neurodegenerative diseases

The first of eight ASOs approved by the US Food and Drug Administration (FDA) for clinical use was antisense DNA oligonucleotide fomivirsen (Vitravene), designed to block replication of cytomegalovirus (CMV) in CMV retinitis. Development of therapeutic ASOs blocking SARS-CoV-2 viral RNA are now in focus.

The introduction of ASOs into the treatment of neurodegenerative diseases was marked by the registration of Nusinersen (Biogen) for therapy of spinal muscular atrophy (SMA). To complement the survival motor neuron 1 (SMN1) deficiency associated with the disease, the therapeutic ASO facilitates the alternative splicing of SMN2 mRNA. Two other ASOs against Huntington's disease entered clinical trials. The first of the investigated ASOs: RG6042 (Roche), targets Huntingtin pre-mRNA and induces its RNase H-mediated degradation. Phase I/II studies proved that RG6042 is well-tolerated, safe, and resulted in a 40-60% decrease in mutant Huntingtin concentration in CSF [24], however, an absence of clinical benefit has resulted in the Phase III study being halted (NCT03761849) (www.clinicaltrials.gov, accessed 2021-09-28). Another approach exploits the presence of single nucleotide polymorphisms (SNPs) associated with CAG expansion, therefore is suitable for treatment of 75-85% of all HD patients. The strength of this approach is selective elimination of only mutant huntingtin without loss of wild-type huntingtin preserving its physiological function. However, the PRECISION-HD1 and 2 trials were both terminated this year for lack of efficacy (www.clinicaltrials.gov; NCT03225833 and NCT03225846 respectively, accessed 2021-10-27), suggesting more work is needed to fully understand the mechanism [25]. Several other ASOs for treatment of neurodegenerative diseases are currently in clinical trials or at preclinical stages of development, as reviewed elsewhere [15,26].

## 5 ASOs regulating mRNA in Alzheimer's disease

ASOs have also been designed for AD therapy but none have yet entered clinical trials. The ASOs in preclinical tests aim mainly at classical targets, described here

### 5.1 Aβ pathology

Several ASOs aimed at lowering levels of toxic Aβ by targeting mRNA for APP or its amyloidogenic processing enzymes. OL-1 was an ASO designed to target the APP mRNA region corresponding to the 17-30 amino acid fragment of Aβ [27]. OL-1 lowered APP expression in the brain of two AD mouse models: transgenic Tg2576(APPswe) and SAMP8 mice which develop Aβ plaques during ageing (SAMP8 spontaneously). OL-1 treated mice, despite concerns raised by the observed shift towards soluble Aβ, were characterised by improved cognitive performance and reduced neuroinflammation [28,29]. Another ASO was tested for splicing-switching to favour APP mRNA lacking exon 17 (exon 15 in mice) which encodes γ-secretase cleavage sites [30]. This ASO caused a reduction in Aβ_42_ levels in the hippocampus of wild type C57BL/6J mice. Tg2576(APPswe) mice were also injected with two ASOs designed to block human APP mRNA translation at γ-secretase or mutated β-secretase cleavage sites [31]. ASO-based β-site targeting resulted in a decreased ratio of cerebral Aβ_40/42_, however no effect was observed for γ-site elimination. ASO directed at APP processing PS1 lowered Aβ-mediated brain oxidative stress as well as improved learning and memory in aged SAMP8 mice [32]. Also, BACE1 mRNA and protein levels were downregulated by another ASO by respectively 90% and 45% in the HEK293 cell line but this ASO awaits validation *in vivo* [33].

*Tau pathology* Despite tau's role in neuronal microtubule assembly and stability, complete abrogation of tau expression did not result in behavioural or neuroanatomical abnormalities in adult mice [34]. This discovery encouraged attempts to reduce tau levels with ASOs for treatment of AD and other tauopathies associated with intraneuronal accumulation of toxic tau [35]. An ASO designed to induce RNase H-mediated degradation of tau mRNA caused a decrease in the level of tau protein in the brain, inhibited hippocampal and neuronal loss, diminished ability for tau aggregate propagation, and extended survival in tauP301S (PS19) mice. Decreases in tau mRNA and protein expression were also observed in non-human primate *Cynomolgus* monkeys [35]. The primary results of phase I/II clinical trials based on this ASO are expected in 2022 (NCT03186989). Another promising ASO has targeted glycogen synthase kinase-3 (GSK-3β), a prominent enzyme phosphorylating tau [36]. Intracerebroventricular administration of this ASO to SAMP8 mice resulted in decreased tau phosphorylation and lower oxidative stress, as well as in improved learning and memory [37]. Similar results were later obtained for peripheral administration of this ASO in TG2576(APPswe) AD mice [38].

*ApoE* ApoE4 isoform is the most significant genetic risk factor for AD (homozygotes have 12 times higher risk for AD). In an APP/PS1 mouse model homozygous for the ApoE4 isoform, administration of an ASO targeting ApoE mRNA decreased APOE protein levels in the brain, however the lowering of Aβ plaque burden was observed only when the treatment began within 24 hours postpartum and not in mature mice [39]. Another approach modulated the splice forms ratio of the ApoE receptor ApoER2, because its splicing is dysregulated in the brains of AD patients and in AD model mice (TgCRND8) [40]. Use of an ASO designed to promote exon 19 inclusion in ApoER2 mRNA improved synaptic function, learning, and memory in mice, however no changes in Aβ levels were reported.

### 5.2 Other targets

Growing evidence highlights the role of neuroinflammation and systemic inflammation in

AD pathogenesis. ASO-mediated depletion of plasminogen, an enzyme involved in proinflammatory reactions, resulted in a decrease in glial activation, Aβ plaque deposition, and neuronal damage in Tg6799 AD model mice [41]. AD pathogenesis is also linked with epigenetic dysregulation driven by DNA methyltransferases (DNMTs) and histone deacetylases (HDAC). Although a detailed mechanism has not yet been elucidated, HDAC2 was shown to correlate with Aβ levels and tau hyperphosphorylation and aggregation in 3xTg-AD and P301L tau mice [42,43]. An ASO targeting HDAC2 mRNA increased memory in an AD mouse model (B6129S F1 hybrid) and modulated cortical and hippocampal expression of signaling proteins implicated in memory formation (ERK1, MHCI, TNF, S100A) [44]. Most of the currently available drugs for AD symptoms inhibit activity of acetylcholinesterase (AChE), but interact also with non-specific targets and produce side effects. Targeting AChE mRNA with ASO in mice treated with intravenous administration of Aβ resulted in improved cognition and memory without significant side effects [45]. This finding highlights the increased specificity of ASO-based therapies over small molecule protein inhibitors.

## 6 Novel ASO paradigm based on miRNA: antagomiRs and miRNA mimics

**Fig. 3:**
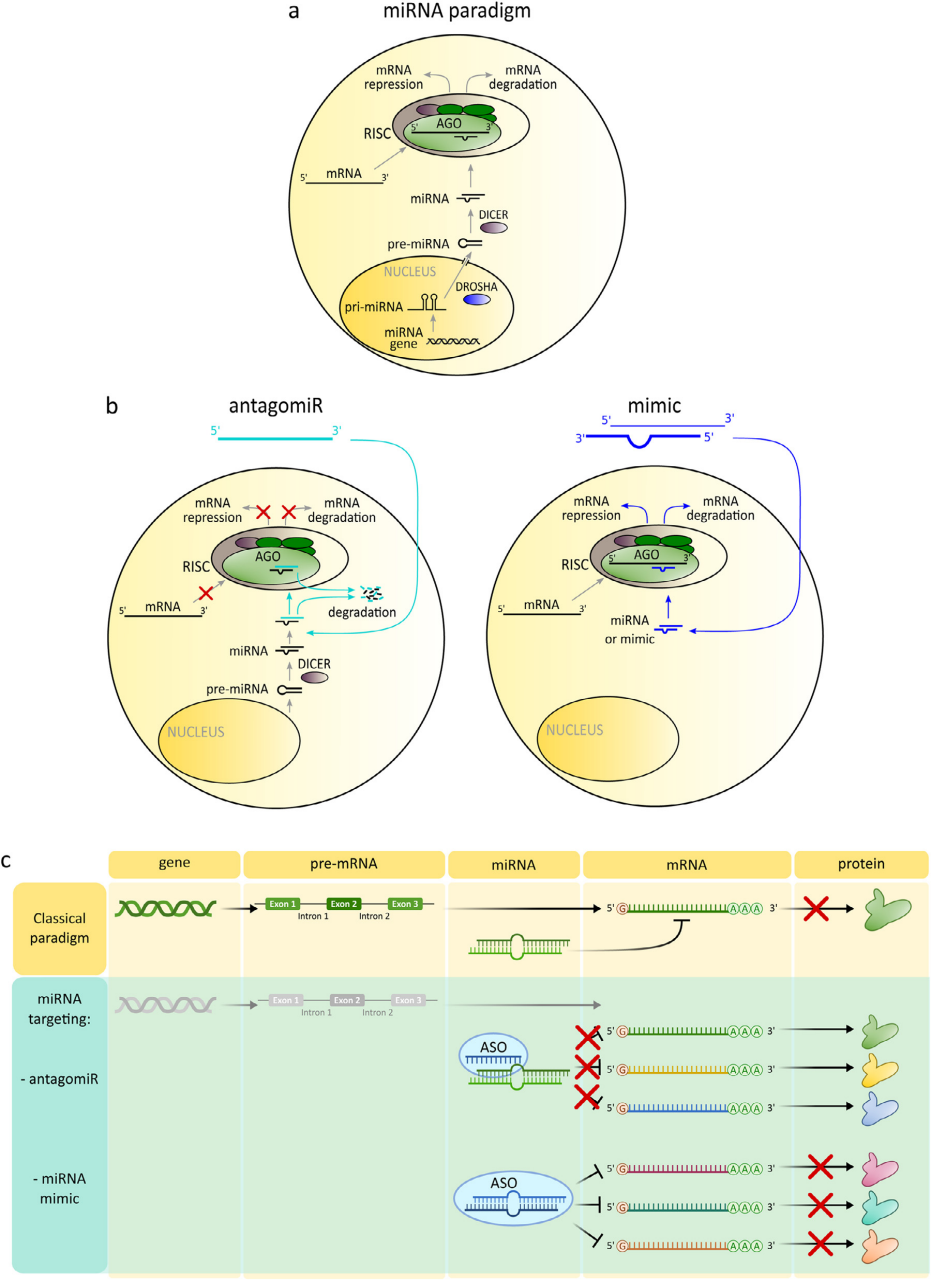
**Therapeutic ASO paradigm based on miRNA.** (a) Scheme of canonical biogenesis of miRNA and RNA interference. Subsequent steps of miRNA processing in the cell result in the epigenetic regulation of mRNA by RNA-induced silencing complex (RISC)**.** (b) Therapeutic ASOs as mimics of endogenous miRNAs and antagomiRs: mechanism of modulation of RNA interference. (c) Multi-targeting mechanisms achieved with miRNA mimics and antagomiRs, resulting respectively in multiple protein up- or downregulation.

From the first evidence in the 1980s, we witnessed a profound increase in the recognition of the regulatory functions of non-coding RNAs (ncRNA), representing over 90% of all translated RNAs. In this context, a new era in the development of ASOs as therapeutics started with the milestone discovery in 1993 of non-coding small regulatory RNAs called microRNAs (miRNAs) and of RNA interference (RNAi), awarded in 2006 with Nobel Prize in Physiology or Medicine [46,47]. RNAi is a process by which small RNA molecules inhibit mRNA translation, providing control of gene expression at the post-transcriptional level. Endogenously, RNAi is mediated by a group of 19 to 24 nucleotide-long miRNAs (miRNAs) and by the RNA-induced silencing complex (RISC), (Fig. 3a).

Two decades of miRNA research has resulted in the discovery of 38 589 mature miRNAs in 271 species registered in miRBase; (http://www.mirbase.org accessed 2021-04-22). The number of mature miRNAs identified in humans approaches 2000 and over 60% of human genes are in scope of their regulation [48]. Under miRNA control are such vital biological processes as development and ageing, and at the cellular level: proliferation, differentiation, DNA repair, apoptosis, and metabolism [49,50]. Since a single miRNA can regulate multiple target mRNAs and a single transcript can be regulated by several miRNAs, the miRNA regulatory network is complex. Importantly, particular miRNAs seem to orchestrate whole signaling pathways, so targeting one miRNA can have profound effects on particular cellular responses [51,52]. These data put miRNAs at centre stage in the epigenetic posttranscriptional regulation landscape and open new perspectives on miRNA-based ASOs as more powerful therapeutics compared with traditional ones. In support of this, miRNAs reprogram somatic cells to induced pluripotent stem cells (iPSC) more effectively and safely than transcription factors, becoming the main tools in development of future iPSC-based therapeutics [53].

The majority of miRNAs are created through canonical biogenesis in a tissue-specific manner [54,55], presented schematically in Fig. 3a and described in the section *6.1. miRNA biogenesis*. Non-canonical biogenesis of miRNAs is also known, and some miRNAs can target a 5′-UTR [56] or play a role in translation enhancement [57,58]. Based on canonical miRNA biogenesis and RNAi, two types of miRNA-based ASOs have been developed: miRNA mimics and miRNA inhibitors called antagomiRs (Fig. 3b). AntagomiRs are synthesised as classical single-stranded oligonucleotides complementary to guide strands of endogenous miRNAs or to pre-miRNA. AntagomiRs are chemically modified for protection against degradation and delivered to the cytoplasm for homologous binding to, and blocking of, the target miRNAs from the interaction with AGO2 protein and entry to RISC. Thus, antagomiRs prevent mRNA degradation or translational repression induced by miRNA. In turn, miRNA mimics are synthesised as 20-22 nucleotide double-stranded RNA oligonucleotides. They mimic duplexes produced by DICER, ready for interaction with AGO2 and integration with RISCs for translational repression of the target mRNA. From this perspective, such ASOs eventually target mRNA and block its translation.

The unique feature of a miRNA mimic or antagomiR is its ability to simultaneously target multiple mRNAs resulting in downregulation or upregulation of multiple proteins (Fig. 3c).

In addition to miRNA-based ASOs, RNAi became the basis for development of double-stranded short interfering siRNA oligonucleotides. These frequently complement the mRNA coding region rather than the 3’-UTR usually targeted by miRNAs. siRNAs have been broadly used in molecular studies for gene knockdown by blocking translation. They can also be delivered using formulation approaches with synthetic non-viral vectors, or by conjugation of ligands (e.g. cholesterol; GalNAc). siRNAs can be introduced into cells not as ASOs but in viral vectors or plasmids as a sequence coding short hairpin RNA (shRNA). In the cells, transcribed shRNAs are processed by DICER and enter RISC like miRNAs. Following the success of siRNA in biomedical research, they are investigated for many therapeutic applications. Currently over 60 siRNA drugs are under investigation or have completed clinical trials as reviewed elsewhere [59,60]. However, this review will focus on ASOs which are antagomiRs or mimics of naturally occurring miRNAs.

### 6.1 miRNA biogenesis

Usually miRNA biogenesis is initiated in the nucleus by Polymerase II or III from genomic sites located outside protein-coding genes or in introns. Polymerase produces long hairpin-like pri-miRNA transcripts further processed by the Microprocessor protein complex, consisting of RNAse III Drosha, and DGCR8 protein, which generates a shorter (∼ 70 nucleotides) hairpin-like pre-miRNA. This pre-miRNA is exported to the cytoplasm by Exportin-5 (XPO-5) and Ran-GTP. After translocation pre-miRNA undergoes cleavage by the endonuclease DICER (RNase III) complexed with double-stranded RNA-binding protein (TRBP). This cleavage separates the loop and releases the stem as a mature double-stranded miRNA duplex with a two-nucleotide-long 3’ overhang on each end. The next step requires the interaction of the duplex with the Argonaute 2 (AGO2) protein, supported by chaperones HSP70/HSP90 and ATP. Upon duplex binding, AGO2 returns to a low-energy conformation releasing one strand of the miRNA duplex known as the passenger strand, which is then degraded. The second, guide strand forms a mature RNA-induced silencing complex (RISC) with AGO2. In the RISC, a single-stranded guide miRNA binds through homologous sequence base pairing of its seed region to the 7-8 nucleotide long site located in the 3′ untranslated region (3′-UTR) of its target mRNA, causing either translational mRNA inhibition or directing mRNA for degradation (Fig. 3a) [55]. Each strand of the miRNA duplex can become a guide strand; both strands possess unique seed sequences defining strand specificity for its target mRNA (strand identities are denoted in miRNA names as -3p, -5p).

### 6.2 miRNA antagomiRs and mimics as novel therapeutics for AD

miRNAs are considered promising therapeutic targets. In 2007 a number of companies were founded focused solely on development of miRNA targeted therapies and their number is still growing, as reviewed elsewhere [61]. Since development of miRNA-targeted ASOs has started relatively recently, most of them are still at the preclinical or even *in vitro* stage. The most advanced of the miRNA-targeted ASOs is Miravirsen developed by Santaris Pharma (now part of Roche) which currently awaits phase II clinical trial results. Miravirsen is a 15-nucleotide antagomiR of miR-122 designed to alleviate hepatitis C virus (HCV) infection. Binding of this RNA virus to miR-122 enables hijacking of the cell machinery and viral propagation whilst protecting it from endogenous nucleases. Miravirsen binds to miR-122 preventing its interaction with viral RNA and virus replication [62]. Indeed, Miravirsen was shown to successfully reduce HCV RNA levels, and was well tolerated [63]. For treatment of AD, multiple targets have been tested with miRNA-based ASOs in preclinical assays, collected in Table 1.

**Table 1:** AntagomiRs and miRNA mimics as potential therapeutics in AD.

	Aβ pathology	Tau pathology	Aβ and tau pathology	Apoptosis and autophagy	Other targets
miR mimics	- miR-124 - miR-188-5p - miR-195 - miR-200b/c	- miR-132-3p - miR-219-5p - miR-483-5p	- miR-16 - miR-31-5p - miR-101	- miR-214-3p - miR-299-5p - miR-326	- miR-101b-3p (HDAC2)
antagomiRs				- miR-34a	- miR-33-5p (ABCA1) - miR-34c-5p (SIRT1) - miR-146a-5p (CFH) - miR-206-3p (BDNF) - miR-937-3p (BRN-4)

*Aβ pathology* Overexpression of miR-200b/c mimics in murine neurons was associated with decreased secretion of toxic Aβ_42_ [64]. Consistently, Tg2576(APPswe) AD mice treated with miR-200b/c mimics proved to be protected from memory loss and learning impairment. Similarly, miR-188-5p counteracted Aβ_42_ synaptic toxicity and related cognition impairment in the 5xFAD(B6SJL) AD mice [65]. Upregulation of two other duplex miRNAs in AD cellular models: miR-195 and miR-124, was able to decrease BACE1 expression and Aβ levels [66,67]

*Tau pathology* Tau mRNA is downregulated by miR-132-3p and levels of this miRNA in brain correlate with tau aggregation and memory impairment in AD patients [68]. 3xTg-AD mice treated with miR-132 mimic presented reduced levels of phosphorylated tau and memory improvement. Moreover, miR-219-5p and miR-483-5p were shown to directly or indirectly regulate levels of pathologically phosphorylated human tau in cellular models, but these ASOs await for verification *in vivo* [69,70].

*Amyloid and tau pathology simultaneously* The strength of miRNA-targeted ASOs is their ability to modulate multiple genes simultaneously. Recently, miR-31-5p was shown to bind to mRNA of both APP and BACE1 resulting in their downregulation [71]. This miRNA was downregulated in AD patients and its overexpression in 3xTg-AD mice led to diminished Aβ deposition and improved cognitive functions. An example of miRNA regulating 3 key proteins of AD pathology is miRNA duplex miR-16, downregulated in patients with sporadic AD. In several cell lines miR-16 overexpression led to lower levels of APP, BACE1, and phosphorylated tau [72] In wild-type mice intracranial delivery of a miR-16 mimic resulted in region-specific lowering of all three targets, and in SAMP8 mouse hippocampus it decreased APP levels [73]. The APP transcript can also be blocked by miR-101 duplex; its mimics reduced APP levels in cell lines [74].

*Apoptosis and autophagy* Another postulated therapeutic option for AD is inhibition of neuronal cell death. Regulation of apoptosis and autophagy in neurons involves miR-299-5p and miR-214-3p, whose levels are decreased in CSF of AD patients [75,76] Model AD mice (APP/PS1 or SAMP8), injected with miR-299-5p or miR-214-3p mimics respectively, were characterised by drops in autophagy and apoptosis as well as improved cognitive performance [75,76] In turn, miR-326 controls the JNK pathway mediating response to various extracellular stress stimuli and acting upstream of apoptosis signaling factors Bax, Bcl-2, and caspase-3 [77] miR-326 mimic treatment of APPswe/PSΔE9 AD mice inhibited neuronal apoptosis, decreased brain levels of Aβ and phospho-tau, and improved cognition. Neuronal apoptosis was also reduced in neuroblastoma cells using miR-34a antagomiRs which prevented miR-34a-mediated downregulation of anti-apoptotic Bcl-2 protein [78].

*Other targets* Downregulated by miR-33-5p, ABCA1 is an enzyme responsible for ApoE lipidation, which in turn is known to affect Aβ metabolism. Administration of a miR-33-5p antagomiR resulted in elevated ABCA1 levels in wild-type C57BL/B6 mice [79] and decreased Aβ level in cortices of APP/PS1 mice [80]. Donepezil, used for alleviating AD symptoms, acts through suppression of neurotrophic BDNF and is suspected to exert its function via interaction with miR-206-3p. Interestingly, this miRNA is upregulated in the cortex of APP/PS1 and APPswe mice, and AD patients [81,82] A miR-206-3p antagomiR injected into cerebral ventricles of APPswe mice or administered intranasally increased BDNF levels, enhanced synaptic density and neurogenesis, and improved memory [82] HDAC2 exerts its regulatory effect on tau via modulation of miR-101b-3p and AMPK. A miR-101b-3p mimic in 3xTg-AD mice resulted in decreased tau phosphorylation and dendritic impairment followed by memory improvement [42]. SIRT1 is the best studied member of the sirtuin family of NAD^+^ dependent deacetylases that regulate cellular responses to stress and are implicated in ageing and related diseases. Elevated cellular sirtuin levels are considered protective and SIRT1 mRNA has two binding sites for miR-34c-5p [83] which was found at high levels in the hippocampus of AD patients and SAMP8 mice. While miR-34c-5p overexpression was associated with learning impairment and reduced SIRT1, an antagomiR was able to rescue learning impairment in APP/PS1 mice. Consistently, miR-34c-5p-targeted antagomiRs improved memory in SAMP8 [83]. BRN-4 is a transcription factor that plays a crucial role in neuronal development and is regulated by miR-937-3p. Mesenchymal stem cells treated with miR-937-3p antagomiR and subsequently transplanted into APP/PS1 mice contributed to reduced Aβ deposition and increased BDNF levels [84]. Of note, many miRNAs have been implicated in neuroinflammation, a process currently seen as a key driver of AD pathology. An example is miR-146a-5p, upregulated in the CSF of AD patients and known to control CFH complement factor of innate immunity [85,86]. Treatment of human neuronal-glial co-cultures with antagomiRs against miR-146a-5p counteracted downregulation of CFH*.* All these miRNA-based ASOs are awaiting further verification as potential drugs for AD.

## 7 Conclusions

Despite huge efforts no approved disease-modifying AD therapies exist. Recent expansion in the understanding of AD pathogenesis beyond the role of Aβ and tau indicates multiple novel therapeutic targets related particularly to neuroinflammation and oxidative stress, which alter dynamically with AD progression. As a multifactorial and progressing disorder, AD is profoundly challenging and requires an appropriate, presently unavailable, therapeutic paradigm. Compared to traditional targeted molecular therapies, ASOs targeted to mRNA or miRNA seem to fulfil such requirements as they can be relatively easily delivered to the brain and they enable multi-targeting, adjusted moreover sequentially to the disease stage. ASOs offer the highest targeting specificity and a diverse spectrum of regulatory possibilities. ASOs directed to mRNA can not only block translation but also enhance it, allowing for either removal of malfunctioning proteins or restoration of properly functioning lost ones. Also, ASOs can uniquely allow selection of protein spliced variants. However, while so far ASOs have been tested for specific targeting of single mRNAs, their main advantage is the capacity for simultaneous regulation of several different transcripts. An additional dimension to this multi-targeting perspective came recently with the discovery of miRNAs. As one miRNA can regulate many proteins, often of the same signalling pathway, miRNA-based ASOs open the possibility for synchronous regulation of entire pathways and even cellular signalling networks. The proof of concept based on naturally existing miRNA-regulated networks can be found in reprogramming of somatic cells to stem cells using only two miRNAs. Such a network effect seems within reach with antagomiRs or mimics of endogenous cellular miRNAs but not with siRNAs which are each designed to one specific mRNA. This new concept of targeting whole signalling pathways with miRNA-based ASOs in AD is further supported given that over 70% of miRNAs in humans are expressed in the brain, and many are involved in the regulation of neuroinflammation and other key pathomechanisms of AD. The realisation of this strategy requires experimental verification of disease-associated miRNA networks. This knowledge is necessary for determining optimal miRNAs as targets of ASOs and for overcoming the risks associated with unwanted interactions and off-target effects. Increasingly accessible methods for high throughput single-cell proteomics and transcriptomics can support achieving this aim.

Preclinical tests demonstrated the regulatory potential of mRNA and miRNA-based ASOs towards classical and novel targets identified in AD pathology, but these approaches are at an early stage and await further validation. These ASOs were tested in a very limited number of AD mouse models. The serious limitations of these mouse lines in reflecting the complexity of human SAD suggests that improved models are required. For instance, employing novel advanced animal and cellular models of SAD, such as iPS-derived human organoids obtained from somatic cells of AD patients, could help select ASOs for clinical trials.

Among ASOs’ advantages for treatment of human brain diseases is the possibility of efficient, low-immunogenic delivery of ASOs to cells in the human brain. While brain delivery of chemically modified ASOs following injection to meninges or intravenously has been shown in rodents [9], this has yet to be emulated in humans, however progress is still being made [87]. Alternative approaches such as transient permeabilisation of the BBB are also being explored [88]. Upon delivery, ASOs trigger a transient effect making it easy to optimise doses, adjust formulations, or discontinue treatment in case of unwanted effects. In order to be viable therapeutic compounds, limitations such as: the physiological and economic effects of continued administration, potential long-term effects vs clearance in the body and the distribution following administration to required sites must be further optimised.

Whilst the majority of current ASOs in the clinic are unencapsulated, the benefits of more specific tissue-/cell-targeting and enhanced cellular uptake should inspire developing novel encapsulation and surface functionalisation technologies. This could also aid the adjustment of the therapeutic ASO levels. Co-administration of ASOs along with well-established drugs is also pursued [89,90].

In summary, ASOs targeted to mRNA and particularly miRNA offer potential for therapy of multifactorial diseases with complex aetiologies, such as AD. Examples of ASO translation to human clinical trials demonstrate that effective therapies are within reach, given further progress in ASO delivery methods and mechanistic understanding of miRNA-mRNA network regulation in AD.

### 7.1 Outstanding Questions

AD, as a multifactorial disease, requires multi-targeted therapy started at the early disease stage and adjusted to different AD stages. This can be achieved with development of ASOs as they allow for multi-targeting and disease-stage-specific therapy.

1 To this aim better elucidation of the AD pathomechanism is required with identification of key master regulators of the complex signalling network underlying AD brain pathology.
2 The search for such key endogenous regulators seems particularly promising among miRNAs in regulatory networks of AD.
3 Development of ASO mimics and antagomiRs is needed to enhance or eliminate respectively the effects of those identified key miRNAs.
4 Further development of administration methods and formulations is required to ensure efficient brain delivery of ASOs at therapeutically meaningful concentrations.
5 Development and refinement of sporadic AD animal models can facilitate preclinical assessment of ASOs in the correct biological context.

### 7.2 Search strategy and selection criteria

We searched PubMed for articles in English from 1^st^ January 1990 to 15^th^ May 2021 using search terms “antisense nucleotides”, “ASO AND neurodegenerative diseases”, “ASO AND Alzheimer's Disease”, “ASO AND microRNA”, “antagomirs”, “microRNA mimics”, “ASO AND microRNA AND Alzheimer's Disease”, “ASO delivery”. In the case of mRNA targeting ASOs only *in vivo*, preclinical or clinical studies were included. The final reference list was generated on the basis of relevance and originality with regard to the topics covered in this Review.

## Declaration of Competing Interest

UW is the coauthor on a patent concerning use of circulating miRNAs as diagnostic biomarkers for early Alzheimer's disease (EP3449009)
